# Matched-pair analysis of [^177^Lu]Lu-PSMA I&T and [^177^Lu]Lu-PSMA-617 in patients with metastatic castration-resistant prostate cancer

**DOI:** 10.1007/s00259-022-05744-6

**Published:** 2022-03-04

**Authors:** Philipp E. Hartrampf, Franz-Xaver Weinzierl, Andreas K. Buck, Steven P. Rowe, Takahiro Higuchi, Anna Katharina Seitz, Hubert Kübler, Andreas Schirbel, Markus Essler, Ralph A. Bundschuh, Rudolf A. Werner

**Affiliations:** 1 Department of Nuclear Medicine, University Hospital Wuerzburg, Oberdürrbacherstraße 6, 97080 Würzburg, Germany; 2 Department of Radiology and Radiological Science, The Russell H Morgan, Johns Hopkins University School of Medicine, 601 N Caroline Str, Baltimore, MD USA; 3 Department of Urology and Paediatric Urology, University Hospital Wuerzburg, Oberdürrbacherstraße 6, 97080 Würzburg, Germany; 4 Department of Nuclear Medicine, University Hospital Bonn, Venusberg-Campus 1, 53127 Bonn, Germany

**Keywords:** PSMA I&T, PSMA-617, Prostate-specific membrane antigen, Prostate cancer, Radioligand therapy, Matched pair

## Abstract

**Background:**

Labelled with lutetium-177, the urea-based small molecules PSMA I&T and PSMA-617 are the two agents most frequently used for radioligand therapy (RLT) in patients with advanced metastatic castration-resistant and prostate-specific membrane antigen (PSMA) expressing prostate cancer (mCRPC). In this matched-pair analysis, we aimed to compare the toxicity and efficacy of both agents for PSMA-directed RLT.

**Materials and methods:**

A total of 110 mCRPC patients from two centres were accrued, 55 individuals treated with [^177^Lu]Lu-PSMA I&T, and a matched cohort of 55 patients treated with [^177^Lu]Lu-PSMA-617. Matching criteria included age at the first cycle, Gleason score, prostate-specific antigen (PSA) values, and previous taxane-based chemotherapy. Using common terminology criteria for adverse events (CTCAE v. 5.0), toxicity profiles were investigated (including bone marrow and renal toxicity). Overall survival (OS) between both groups was compared.

**Results:**

Toxicity assessment revealed grade III anaemia in a single patient (1.8%) for [^177^Lu]Lu-PSMA I&T and five (9.1%) for [^177^Lu]Lu-PSMA-617. In addition, one (1.9%) grade III thrombopenia for [^177^Lu]Lu-PSMA-617 was recorded. Apart from that, no other grade III/IV toxicities were present. A median OS of 12 months for patients treated with [^177^Lu]Lu-PSMA I&T did not differ significantly when compared to patients treated with [^177^Lu]Lu-PSMA-617 (median OS, 13 months; *P* = 0.89).

**Conclusion:**

In this matched-pair analysis of patients receiving one of the two agents most frequently applied for PSMA RLT, the rate of clinically relevant toxicities was low for both compounds. In addition, no relevant differences for OS were observed.

## Introduction

As the second most frequent malignancy in men worldwide, the annual incidence of prostate cancer (PC) is 1.4 million and accounts for > 375,000 deaths [1]. The transition from an androgen-dependent to a castration-resistant phenotype poses challenges for treating clinicians, especially in widespread diseases. Recent years have witnessed the introduction of novel therapeutic regimens for metastatic castration-resistant prostate cancer (mCRPC), including androgen receptor signalling inhibitors [2, 3] chemotherapeutics [4–6], and prostate-specific membrane antigen (PSMA)-directed radioligand therapies (RLT), which are typically carried out with small-molecule, urea-based agents linked to the ß-emitting radionuclide lutetium-177 [7]. In this regard, recent prospective trials have shown remarkable outcomes after PSMA RLT in patients with mCRPC [8–11]. For instance, the VISION trial reported a substantial prolongation of overall survival (OS) when compared to current standard-of-care therapy, along with an objective response rate in approximately half of the patients [12].

**Fig. 1:**
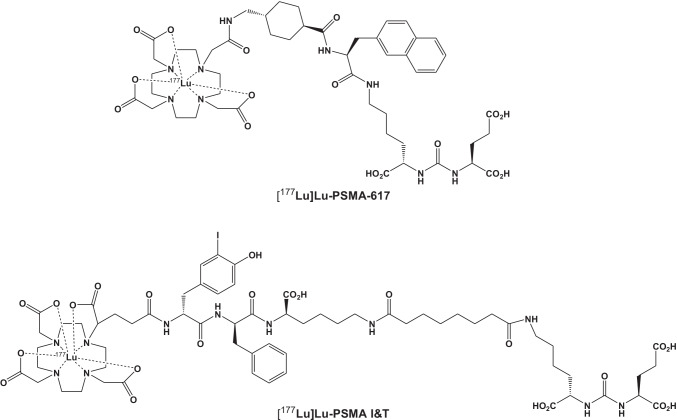
Structures of [^177^Lu]Lu-PSMA-617 and [^177^Lu]Lu-PSMA I&T, demonstrating underlying differences of both agents. Only the urea-binding motif of the two peptides is identical. For [^177^Lu]Lu-PSMA-617, the peptide is linked to a DOTA chelator, while for [^177^Lu]Lu-PSMA I&T, a DOTAGA chelator is used

To date, PSMA-targeted RLT is mainly conducted with the two different agents, [^177^Lu]Lu-PSMA I&T and [^177^Lu]Lu-PSMA-617 [7]. As shown in Fig. 1, the chemical structures of the two labelled peptides for PSMA-directed RLT show significant differences. Only the urea-binding motif of the two peptides is identical, but the majority of the two molecules show no structural similarity. On closer inspection, even the chelators are different: while in [^177^Lu]Lu-PSMA-617, the peptide is linked to a DOTA chelator [13], in [^177^Lu]Lu-PSMA I&T, the related DOTAGA chelator is used [14]. Of note, a recent dosimetry study reported on comparable mean absorbed tumour doses for both compounds [15]. In a preclinical setting, [^177^Lu]Lu-PSMA-617 seems to be superior when compared to [^177^Lu]Lu-PSMA I&T [16]. Head-to-head comparisons in patients, however, are still lacking [7]. In this two-centre, matched-pair analysis, we compared the toxicity and efficacy of [^177^Lu]Lu-PSMA I&T and [^177^Lu]Lu-PSMA-617 for treatment of patients with mCRPC.

## Materials and methods

### Patient cohort

This retrospective study consisted of 55 patients who were treated with [^177^Lu]Lu-PSMA I&T for mCRPC at the University Hospital Würzburg (Germany) and received at least two cycles of RLT. The matched cohort included 55 patients with mCRPC treated with at least two cycles of [^177^Lu]Lu-PSMA-617 at the University Hospital Bonn (Germany). Matching criteria included age at the first cycle, Gleason score (categories: 6–7, 8–10, or unknown, [17]), baseline PSA values (0–50 ng/mL, 50–200 ng/ml, 200–600 ng/ml, and > 600 ng/mL), and previous taxane-based chemotherapies (CTx). Approvals were waived by the local Ethics Committees of Bonn and Würzburg due to the retrospective nature of this analysis (20210422 04).

### Treatment protocol

Standardized institutional protocols for the execution of RLT were applied. In-house labelling was carried out for [^177^Lu]-labelled PSMA ligands ([^177^Lu]Lu-PSMA I&T (Würzburg) and [^177^Lu]Lu-PSMA-617 (Bonn)). Radiosynthesis of [^177^Lu]Lu-PSMA I&T and [^177^Lu]Lu-PSMA-617 have been described elsewhere [18, 19]. Identical treatment protocols at both study sites included administration of approximately 6.0 GBq of the PSMA ligand every 8 weeks with up to a maximum of 8 cycles, depending on the response to treatment [18, 19].

### Toxicity and efficacy assessment

Standard blood collection included prostate-specific antigen (PSA) values, leukocytes, haemoglobin, platelets, creatinine, lactate dehydrogenase, aspartate aminotransferase, gamma-glutamyltransferase, and alkaline phosphatase. By comparing with baseline parameters, toxicity was assessed at the last available cycle. Changes in blood cell counts and creatinine levels were also graded according to the Common Terminology Criteria for Adverse Events (CTCAE v. 5.0) [20]. Efficacy was assessed by comparing OS between both cohorts.

### Statistical analysis

Statistical analyses were performed using GraphPad Prism version 9.3.0 (GraphPad Software, San Diego, CA, USA). Descriptive data (including OS) are presented as median and range in parentheses. Since not all parameters were normally distributed (Shapiro–Wilk test), comparisons between the two cohorts were performed using Wilcoxon signed-rank test. For comparison of dichotomous variables, Fisher’s exact test was applied. OS was analysed using Kaplan–Meier curves and log-rank comparison. A *P*-value less than 0.05 was considered statistically significant.

## Results

### Patients’ characteristics were comparable for both cohorts

Overall, the median patient age at the first cycle of therapy was 71 (46–84) years for [^177^Lu]Lu-PSMA I&T and 70 (43–86) years for [^177^Lu]Lu-PSMA-617. The median Gleason score was 9 in both cohorts. For baseline PSA levels, no significant differences between the groups were observed ([^177^Lu]Lu-PSMA I&T: 168.0 (5.0–3130) ng/ml, [^177^Lu]Lu-PSMA-617: 145 (3.6–2360) ng/ml; *P* = 0.16). In addition, serum chemistry, blood counts, and previous therapies also did not differ between both groups. For preexisting comorbidities, only arterial hypertension differed significantly between both groups ([^177^Lu]Lu-PSMA I&T: 56.4%, [^177^Lu]Lu-PSMA-617: 27.3%; *P* = 0.004). In total, patients received a median of three RLT cycles of either [^177^Lu]Lu-PSMA I&T or [^177^Lu]Lu-PSMA-617 (*P* = 0.77). As such, neither cumulative administered activity ([^177^Lu]Lu-PSMA I&T, 18.0 GBq; [^177^Lu]Lu-PSMA-617, 19.0 GBq), nor median activity per cycle ([^177^Lu]Lu-PSMA I&T, 6.0 GBq; [^177^Lu]Lu-PSMA-617, 6.2 GBq) differed significantly (*P* > 0.05, respectively; Table 1).

**Table 1:** Baseline patient characteristics

	[^177^Lu]Lu- PSMA I&T	[^177^Lu]Lu-PSMA-617	*P*-value
***Clinical variables***			
Age at first cycle of PSMA RLT (years)#	71 (46–84)	70 (43–86)	0.89
Time period between initial diagnosis and 1st RLT (months)	64.0 (9–246)	63 (4–241)	0.37
Treatment cycles per patient	3 (2–8)	3 (2–8)	0.77
Cumulative activity (GBq)	18.0 (10.4–49.2)	19.0 (7.9–45.8)	0.21
Activity per cycle (GBq)	6.0 (3.9–6.8)	6.2 (3.9–9.6)	0.11
Gleason score#	9 (6–10)	9 (6–10)	1.0
**Baseline laboratory values**			
PSA (ng/ml)#	168 (5.0–3130)	145 (3.6–2360)	0.16
LDH (37 °C U/l)	269 (118–1105)	274 (147–2582)	0.13
Leukocytes (Tsd/µl)	6.0 (2.7–15.3)	6.4 (1.5–13.8)	0.46
Haemoglobin (g/dl)	11.5 (8.2–16.1)	11.6 (7.9–13.8)	0.34
Platelets (Tsd/µl)	245.0 (76.0–590)	226.0 (45.0–562)	0.27
Creatinine (mg/dl)	0.92 (0.60–1.92)	0.82 (0.48–2.12)	0.08
AST (37 °C U/l)	28.0 (15.0–62.0)	28.0 (14.0–114.0)	0.70
Gamma-GT (37 °C U/l)	32.1 (12.8–307.1)	33.0 (12.0–1130)	0.48
AP (37 °C U/l)	130 (31.0–1499)	139 (45.0–982)	0.65
***Previous Treatments (%)***			
Radical prostatectomy	40.0	41.8	1.0
Primary radiation therapy to the prostate	16.4	18.2	1.0
Adjuvant radiation therapy	21.8	14.6	0.46
Salvage radiation therapy	18.2	9.1	0.27
Antihormonal treatment	100	100	1.0
Second generation antihormonal treatment (Abiraterone /Enzalutamide)	90.9	89.1	1.0
Chemotherapy#	72.4	72.4	1.0
**Comorbidities (%)**			
Arterial hypertension	56.4	27.3	0.004
Preexisting renal disease	18.2	9.1	0.27
Diabetes mellitus	16.4	3.6	0.05

Abbreviations: *PSA*, prostate specific antigen; *LDH*, lactate dehydrogenase; *AST*, aspartate aminotransferase; *GammaGT*, gamma-glutamyltransferase; *AP*, alkaline phosphatase

^#^ = matching criteria

### Toxicity profiles revealed no relevant differences between both agents

**Fig. 2:**
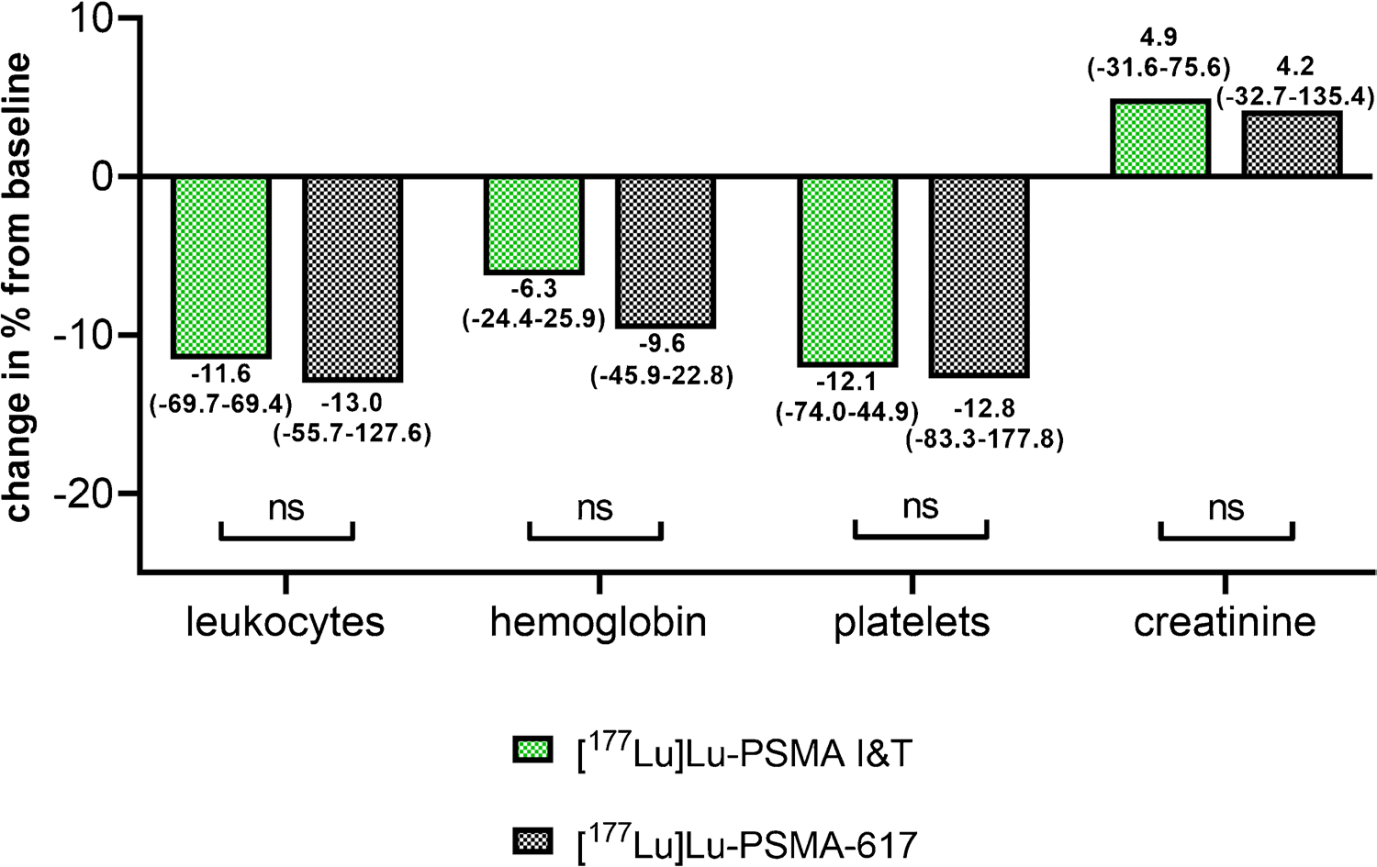
Relative changes in leukocyte counts, platelet counts, and haemoglobin as well as creatinine at the last available cycle of radioligand therapy ([^177^Lu]Lu-PSMA I&T in green, [^177^Lu]Lu-PSMA-617 in black). No significant differences were recorded. Data are presented as median with a range in brackets

**Fig. 3:**
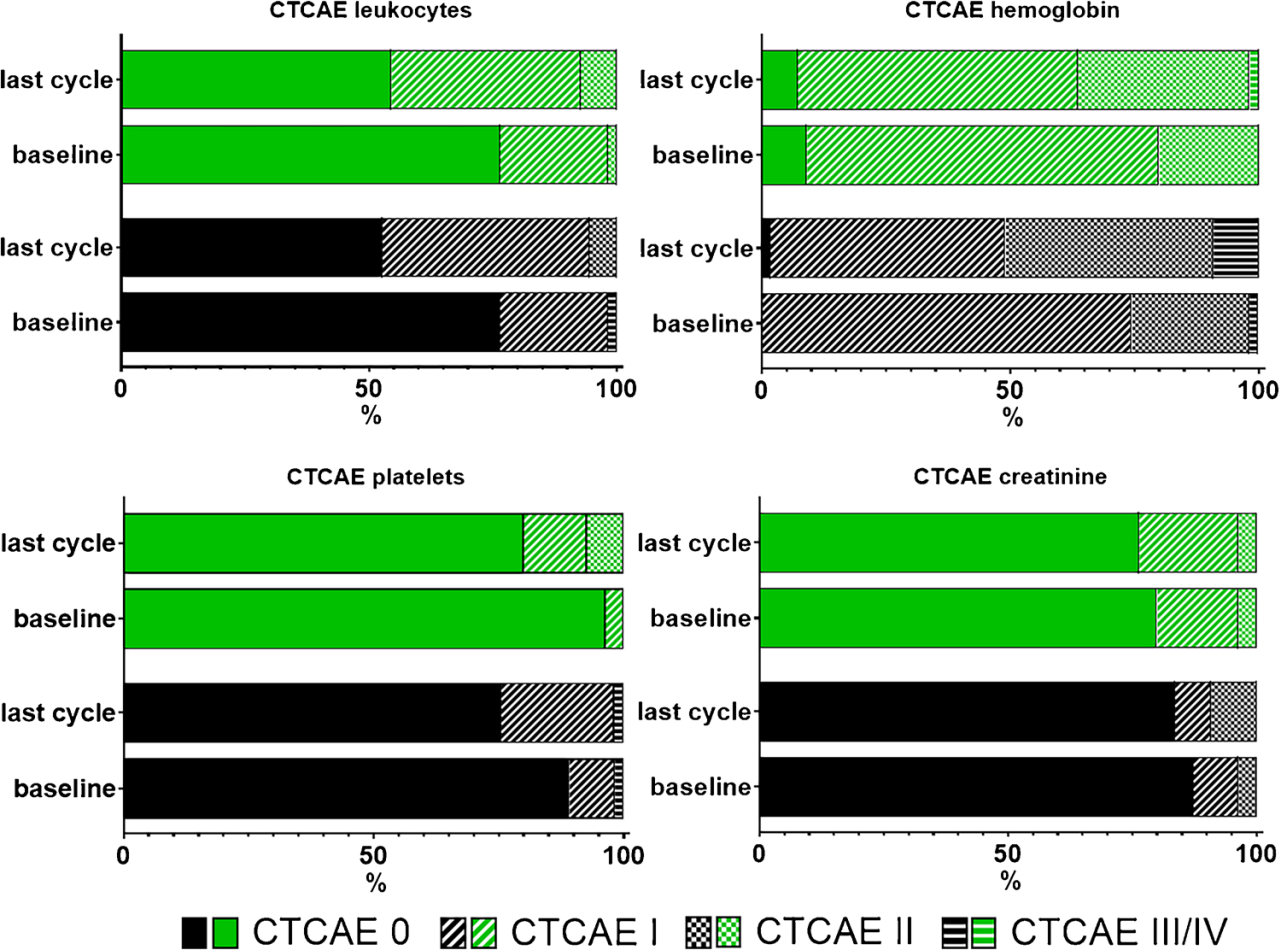
Classifications according to CTCAE version 5.0 for leukocytes, haemoglobin, platelets, and creatinine in percentages for patients treated with [^177^Lu]Lu-PSMA I&T (green) and [^177^Lu]Lu-PSMA-617 (black) at baseline and at the last available cycle. Horizontal lines indicate Grade ≥ III toxicities. For haemoglobin, one grade III toxicity occurred in the [^177^Lu]Lu-PSMA I&T cohort. For [^177^Lu]Lu-PSMA-617, there were five grade III toxicities for haemoglobin and one grade III toxicity for platelets after the last cycle. At baseline, however, one event for haemoglobin and platelets were already recorded for [^177^Lu]Lu-PSMA-617 in different patients. Thus, for this agent, the total number of toxicities in this cohort increased only by four after the last cycle for haemoglobin and remained stable for platelets. No other grade III/IV toxicities occurred for both compounds

During follow-up, patients treated with [^177^Lu]Lu-PSMA I&T demonstrated a median decrease of leukocytes, haemoglobin, and platelets of 11.6%, 6.3%, and 12.1%, respectively. For [^177^Lu]Lu-PSMA-617, comparable respective declines of 13.0%, 9.6%, and 12.8% were recorded. No significant difference was reached for a decrease between both agents. Creatinine levels increased slightly in both cohorts ([^177^Lu]Lu-PSMA I&T, 4.9%; [^177^Lu]Lu-PSMA-617, 4.2%; n.s.; Fig. 2). Compared to baseline, only one grade III anaemia (1.8%) was observed in the [^177^Lu]Lu-PSMA I&T cohort and five grade III anaemia (9.1%) in the [^177^Lu]Lu-PSMA-617 cohort. For the latter agent, there was also one grade III (1.9%) toxicity for platelets after the last cycle. Apart from that, no further grade III/IV toxicities were recorded for either agent (Fig. 3, Table 2).

**Table 2:** Classifications according to CTCAE vers. 5.0 for leukocytes, haemoglobin, platelets, and creatinine for patients treated with [^177^Lu]Lu-PSMA I&T and [^177^Lu]Lu-PSMA-617 at baseline and after the last available cycle. *For [^177^Lu]Lu-PSMA-617, blood values were collected, with only platelets missing in 2 subjects. ^#^For [^177^Lu]Lu-PSMA-617, there were five grade III toxicities for haemoglobin and one grade III toxicity for platelets after the last cycle. At baseline, however, one event for each blood value was already recorded, and thus, the total number of toxicities in this cohort only increased by four after the last cycle for haemoglobin and remained stable for platelets. No other grade III/IV toxicities occurred for both compounds

		[^177^Lu]Lu-PSMA I&T					[^177^Lu]Lu-PSMA-617				
		Grade 0	Grade I	Grade II	Grade III	Grade IV	Grade 0	Grade I	Grade II	Grade III	Grade IV
Leukocytes	Baseline	42/55 (76.4%)	12/55 (21.8%)	1/55 (1.8%)	0.0	0.0	42/55 (76.4%)	12/55 (21.8%)	0.0	1/55 (1.8%)	0.0
	Last cycle	30/55 (54.5%)	21/55 (38.2%)	4/55 (7.3%)	0.0	0.0	29/55 (52.7%)	23/55 (41.8%)	3/55 (5.5%)	0.0	0.0
Haemoglobin	Baseline	5/55 (9.1%)	39/55 (70.9%)	11/55 (20.0%)	0.0	0.0	0.0	41/55 (74.5%)	13/55 (23.6%)	1/55 (1.8%)	0.0
	Last cycle	4/55 (7.3%)	31/55 (56.4%)	19/55 (34.5%)	1/55 (1.8%)	0.0	1/55 (1.8%)	26/55 (47.3%)	23/55 (41.8%)	5/55 (9.1%)^#^	0.0
Platelets	Baseline	53/55 (96.4%)	2/55 (3.6%)	0.0	0.0	0.0	49/55 (89.1%)	5/55 (9.1%)	0.0	1/55 (1.8%)	0.0
	Last cycle*	44/55 (80.0%)	7/55 (12.7%)	4/55 (7.3%)	0.0	0.0	40/53 (75.5%)	12/53 (22.6%)	0.0	1/53 (1.9%)^#^	0.0
Creatinine	Baseline	44/55 (80.0%)	9/55 (16.4%)	2/55 (3.6%)	0.0	0.0	48/55 (87.3%)	5/55 (9.1%)	2/55 (3.6%)	0.0	0.0
	Last cycle	42/55 (76.4%)	11/55 (20.0%)	2/55 (3.6%)	0.0	0.0	46/55 (83.6%)	4/55 (7.3%)	5/55 (9.1%)	0.0	0.0

### Overall survival is also comparable for both agents

**Fig. 4:**
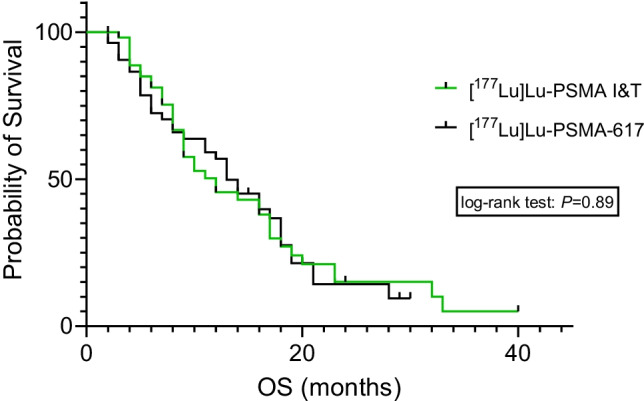
Kaplan–Meier curves of median overall survival (OS) of patients treated with [^177^Lu]Lu-PSMA I&T (median OS 12.0 months, green) and [^177^Lu]Lu-PSMA-617 (median OS 13.0 months, black). Log-rank test showed no significant difference (*P* = 0.89)

Median OS was 12 months for patients treated with [^177^Lu]Lu-PSMA I&T and 13 months for patients treated with [^177^Lu]Lu-PSMA-617 (*P* = 0.89; Fig. 4).

## Discussion

In this matched-pair analysis, we investigated the two most commonly applied urea-based, small-molecule inhibitors of PSMA, namely PSMA-617 and PSMA I&T. We carefully matched patients based on age, Gleason score, and PSA levels. Pre-therapeutic CTx has also been suggested to have a relevant impact on outcome [21] and, thus, we also matched according to this previous treatment regimen. After having controlled for such key variables, toxicity assessments revealed low rates of side effects, with one grade III anaemia for [^177^Lu]Lu-PSMA I&T and five grade III anaemia as well as one grade III thrombopenia for [^177^Lu]Lu-PSMA-617. Moreover, OS for [^177^Lu]Lu-PSMA-617 and [^177^Lu]Lu-PSMA I&T did not significantly differ.

Multiple prospective trials have reported a favourable outcome for [^177^Lu]Lu-PSMA-617, including prolonged OS when compared to standard-of-care [12], and pain reduction in patients with disease progression after conventional treatment [9]. As the second most commonly used RLT agent [7], [^177^Lu]Lu-PSMA I&T has been utilized in retrospective studies demonstrating beneficial outcomes in heavily pre-treated mCRPC patients [22]. Results of a currently recruiting, a prospective clinical trial investigating the performance of this agent as a last-line option will be announced in due course [23]. Despite the encouraging results for both radiopharmaceuticals, head-to-head comparisons are still scarce [7]. In this matched-pair analysis focusing on outcome and toxicity, respective Kaplan–Meier curves for OS revealed no significant differences between patients treated with [^177^Lu]Lu-PSMA I&T (median 12 months) and [^177^Lu]Lu-PSMA-617 (median 13 months). This is in line with a recent dosimetry study comparing both ^177^Lu-labelled, PSMA-directed small molecules. Although no matching was conducted, [^177^Lu]Lu-PSMA I&T had increased initial uptake in metastatic sites of disease, while the residency time and the effective half-life was longer for [^177^Lu]Lu-PSMA-617. Nonetheless, those findings had no relevant impact on the mean absorbed tumour doses ([^177^Lu]Lu-PSMA I&T: 5.8 Gy/GBq, [^177^Lu]Lu-PSMA-617: 5.9 Gy/GBq) [15], which may explain comparable outcome benefits for both agents observed in our study.

No grade III/IV renal toxicities occurred during follow-up. For hematotoxicity, we observed only one patient with new grade III anaemia for [^177^Lu]Lu-PSMA I&T and five patients with new grade III anaemia for [^177^Lu]Lu-PSMA-617. For [^177^Lu]Lu-PSMA-617, there was also one grade III toxicity for platelets. Apart from that, no further grade III/IV toxicities were recorded for either agent. Taken together, this study corroborates previous reports investigating side effects in patients treated with [^177^Lu]Lu-PSMA-617 [7, 10–12, 24] or [^177^Lu]Lu-PSMA I&T [25], which also reported promising safety profiles for both agents. As such, the treating physician can have certainty that both [^177^Lu]Lu-PSMA-617 and [^177^Lu]Lu-PSMA I&T achieve equivalent outcome benefits along with comparable low toxicity profiles.

For PSMA RLT, the structures of the two labelled peptides show relevant differences (Fig. 1) [13, 14]. Furthermore, murine studies performing head-to-head comparisons with both radiotracers demonstrated more favourable biodistribution for [^177^Lu]Lu-PSMA-617 [16]. In light of these preclinical findings and the structural variances among both agents, the similar efficacy and toxicity of the two radiopharmaceuticals were not necessarily expected.

This study has limitations. Striving for an accurate matching, and despite including a total of 110 mCRPC patients, the sample size was rather small. The grouping of patients by original Gleason score categories (i.e. Gleason 6–7 and Gleason 8–10) allowed for adequate matching [17]. Nonetheless, future studies are needed to compare both agents in an even more controlled environment, preferably in a prospective setting. In this regard, such efforts may then adjust for further important variables, including dosimetry aspects, biodistribution, radiotracer preparation, and logistics. Moreover, compared to the VISION trial, which reported on an OS of 15.3 months for [^177^Lu]Lu-PSMA-617 after a median of five cycles of RLT [12], our patients underwent only three cycles of treatment, thereby explaining our shorter OS of only 13 months for this agent. As such, we did not follow current treatment standards as defined by the VISION trial, and future studies should also include patients with a higher number of cycles [12].

## Conclusions

Investigating the most commonly used agents for PSMA-targeted RLT, [^177^Lu]Lu-PSMA I&T and [^177^Lu]Lu-PSMA-617, this matched-pair analysis demonstrates that toxicity profiles of both compounds are comparable, with very low rates of clinically relevant toxicities. In addition, both radiopharmaceuticals are similarly effective in patients with mCRPC.

## Data Availability

Detailed information about the image analysis or the overall survivals of the subjects presented in this study is available on reasonable request from the corresponding author.
